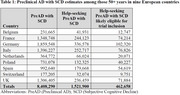# Estimates of preclinical AD with subjective cognitive decline in nine major European countries

**DOI:** 10.1002/alz70859_103039

**Published:** 2025-12-25

**Authors:** Cai Gillis, Shardae Showell, Kristen Lu, Nancy N Maserejian

**Affiliations:** ^1^ Biogen, Cambridge, MA USA

## Abstract

**Background:**

Alzheimer’s disease (AD) begins with a preclinical period of accumulating pathologic biomarkers prior to onset of objective cognitive impairment (CI). Although clinical trials are increasingly targeting this earlier stage, recruitment of preclinical AD (preAD) individuals is challenging due to a lack of appropriate tools to triage presymptomatic patients. PreAD persons with subjective cognitive decline (SCD) may represent a more identifiable pool of participants given that they report SCD but do not yet have overt CI. Here we estimate amyloid‐positive (Aβ+) individuals with SCD in nine European countries among those 50+ years with considerations for help‐seeking and trial eligibility.

**Method:**

We first estimated individuals 50+ years with SCD by applying age‐stratified estimates of SCD prevalence to 2025 United Nations population data for each of the countries examined (Belgium, France, Germany, Italy, Netherlands, Poland, Spain, Switzerland, United Kingdom). Next, we applied age‐stratified estimates of Aβ+ probability from the Amyloid Biomarker Study among those with SCD. We then applied estimates from a European study of help‐seeking for SCD symptoms to determine the number of preAD likely to speak to a healthcare professional (HCP) regarding their cognitive symptoms. Finally, we estimated those potentially eligible for inclusion in a preAD trial by applying estimates of relevant screen‐failure criteria from a trial in a preAD population.

**Result:**

Across nine European countries, approximately 8.4 million people 50+ years with SCD are potentially preAD (Table 1). Estimates ranged from ∼177k in Switzerland to ∼1.9 million in Germany. Of those with preAD, approximately 1.5 million likely have spoken with an HCP regarding their symptoms. When considering potential eligibility for a clinical trial of preAD, an estimated 460k of those who have spoken to an HCP would likely qualify for trial inclusion, ranging from approximately 10k in Switzerland to 102k in Germany.

**Conclusion:**

Of the ∼8 million individuals in nine European countries with preAD and SCD symptoms, only 5.5% would likely be identifiable and eligible for a trial of preAD individuals. Future work should incorporate tau pathology and *APOE ε4* status into estimates. Accelerating development of biomarkers and other diagnostic tools is important to identifying additional preAD trial participants.